# Comparing Machine Learning-Based Crime Predictions Across Micro-Geographic Units: Street Segments, Rectangular Grids, and Hexagonal Grids

**DOI:** 10.1007/s12061-025-09683-1

**Published:** 2025-06-25

**Authors:** Robin Khalfa, Wim Hardyns

**Affiliations:** 1 Ghent University, Ghent, Belgium; 2 University of Antwerp, Antwerp, Belgium

**Keywords:** Crime prediction, Micro-geographic units, Street segments, Grid cells, Machine learning, Spatial analysis

## Abstract

This study examines the potential of alternative micro-geographic units of analysis compared to the widely used rectangular grid for predicting (monthly) micro-geographic crime risks using a machine learning approach. Specifically, this study compares the prediction performance of machine learning models (XGBoost) in deriving monthly micro-geographic risk predictions for three crime types across rectangular and hexagonal grids of varying resolutions, as well as street segments, using the hit rate, precision, and F1-score as key performance measures. Police-registered data on residential burglary, aggressive theft, and battery (2013–2018), along with environmental and seasonal data on crime predictors were used to train the models and evaluate their performance across different units of analysis and performance measures. Results show that street segments generally achieve higher hit rates compared to grid-based units, but only marginally when compared to high-resolution grids (0.0025 km²). This study thus finds no clear advantage of street segments over small grids in terms of model hit rate. In addition, using street segments and small grids comes at the cost of lower model precision, resulting in more false positive predictions. Grids with resolutions from 0.04 km² to 0.25 km² offer a more balanced performance. Further, no substantial differences were found between rectangular and hexagonal grids, indicating grid shape does not affect prediction performance. Future work should explore how model performance should be defined and operationalised within the context predicting crime risks at specific micro-geographic levels and what the implications are of employing specific micro-geographic units of analysis within the context of crime prevention.

**Supplementary Information:**

The online version contains supplementary material available at https://doi.org/10.1007/s12061-025-09683-1.

## Introduction and Background

Spatiotemporal crime prediction has become a central field of inquiry within environmental criminology and the criminology of place, encompassing the use of historical data and statistical (learning) methods to predict where a crime is more likely to occur in the (near) future (Hardyns & Rummens, 2018; Hart, 2020; Kadar et al., 2019; Kronkvist et al., 2025; Rosser et al., 2017; Rummens & Hardyns, 2020; Wheeler & Steenbeek, 2021). This development reflects a broader shift from static spatial crime analysis toward more dynamic and proactive approaches to spatiotemporal crime modelling (Bowers et al., 2004; Johnson & Bowers, 2004; Rosser et al., 2017). Central to this development lies the empirical observation that crime not only concentrates in specific places (Eck & Weisburd, 1995; Evans & Herbert, 1989; Johnson, 2010; Sherman & Weisburd, 1995; Sherman et al., 1989; Weisburd, 2015; Weisburd et al., 2009) but also at specific moments in time and that the intensity and risk of crime in specific places is temporally variable, particularly on the short-term (Brantingham & Brantingham, 2013; Prieto-Curiel, 2023). In this regard, it is argued that spatiotemporal crime prediction models hold the potential to enable a more efficient and effective allocation of (proactive) crime prevention resources, allowing to concentrate prevention resources in or near areas where crime is most likely to occur in the (near) future (Rosser et al., 2017; Wheeler & Steenbeek, 2021).

Despite the clear spatial nature underpinning a spatiotemporal crime prediction task, however, only limited scholarly attention has addressed how the selection and configuration of spatial units of analysis influence crime prediction outcomes (Rosser et al., 2017). Within the criminology of place, spatial units of crime analysis have predominantly been conceptualised as micro-geographic units of analysis (Johnson, 2010; Weisburd, 2015; Weisburd et al., 2009). Micro-geographic units, in this context, refer to small-scale spatial divisions of a specific study setting that serve as analytical entities for examining spatial crime patterns (Weisburd, 2015; Weisburd et al., 2024). While these may theoretically include a wide range of spatial units such as individual addresses, street segments, intersections, blocks, or various types of grid cells, the majority of both academic research and practical applications on spatiotemporal crime prediction have defaulted to generating crime predictions using rectangular (i.e., squared) grid cells of varying resolution as predominant micro-geographic unit of analysis (see e.g., Kounadi et al., 2020 for an overview; Rosser et al., 2017).

Although environmental criminologists generally posit that the choice of the spatial unit of crime analysis should be guided by theoretical and methodological motivations, data availability, and specific research questions (Brantingham et al., 2009; Eck & Weisburd, 1995; Johnson et al., 2010; Weisburd et al., 2009), the widespread adoption of rectangular grids in crime prediction often lacks explicit justification. Hence, while there is clearly no universal ‘golden standard’ for selecting a micro-geographic unit of analysis in crime prediction, this widespread use of rectangular micro-spatial grids warrants critical examination, particularly if alternative units may offer greater analytical or practical relevance (Rosser et al., 2017). However, it remains currently unclear whether alternative spatial units can achieve comparable or superior prediction performance. If a particular unit consistently enhances crime predictions within a given context, the choice of using a specific micro-geographic prediction unit should be considered a key methodological decision, alongside the presence or absence of other theoretical, methodological and practical assumptions and considerations.

The primary objective of this study is to empirically assess the potential of two specific alternative micro-geographic units of analysis, namely street segments and hexagonal shaped grids, compared to rectangular grids in the context of predicting monthly crime risks using a machine learning modelling approach. Our focus on these three specific micro-geographic units (street segments, rectangular grids, and hexagonal grids) is deliberate and justified by both the relevance of street network and grid-based approaches in contemporary analyses of spatial phenomena such as crime and our aim to systematically extend prior comparative research (e.g., Rosser et al., 2017; Rummens & Hardyns, 2021; Wheeler & Steenbeek, 2021). In addition, the choice to employ machine learning modelling can similarly be justified, as machine learning methods have gained increasing prominence in crime prediction due to their capacity to model complex, non-linear relationships and interactions within high-dimensional spatiotemporal data, frequently outperforming traditional statistical techniques such as risk terrain modelling or near repeat analysis (e.g., Ohyama & Amemiya, 2018; Rummens & Hardyns, 2020; Wheeler & Steenbeek, 2021). In this paper, the use of machine learning is not positioned as a critique of traditional crime prediction approaches per se, but rather as a pragmatic choice to handle complex, high-dimensional spatiotemporal data at different micro-geographic units of analysis.

Within this context, the present study makes two key contributions to the crime prediction literature:

First, this study compares the performance of machine learning models in deriving (monthly) predictions for three specific types of crime (residential burglary, aggressive theft and battery) across street segments and rectangular grids of varying grid resolutions. In doing so, this study extends the work of Rosser et al. (2017), who showed that street segments outperformed 150 × 150 m rectangular grids in predicting burglary hot spots using a kernel-based prediction model. Their argument was that street segments more accurately reflect how crime and police activity concentrate along the street network, whereas grid cells may intersect physical barriers or include disconnected spaces (Bowers & Johnson, 2014; Johnson & Bowers, 2010; Weisburd et al., 2012). In addition, from a theoretical perspective, the street network is considered a more meaningful unit of analysis as crime risk is shaped by street network characteristics that influence offender awareness spaces, and criminal activity tends to spread along connected streets rather than uniformly in all directions as implied by grid-based approaches. While valuable, the contribution of Rosser et al. (2017) focused on a single crime type, a fixed grid size, one prediction method, and only one performance measure (hit rate). Our study expands this by incorporating multiple crime types, a machine learning framework, varying grid resolutions, and a broader set of evaluation metrics (hit rate, precision, F1-score), offering a more comprehensive assessment.

Second, we assess the potential of hexagonal grids in this context, an approach still largely absent from crime prediction research. Prior research suggests hexagonal grids may outperform rectangular grids in spatial analyses due to their reduced edge effects, uniform shape, and ability to better capture curved spatial patterns (Birch et al., 2007; Harinam et al., 2022; Hoet, 2023). In addition, their uniform edge contact and equidistant centroids are argued to facilitate more accurate neighbour detection and connectivity analysis. These properties may support more accurate spatial modelling, yet their performance relative to standard rectangular grids and street segments remains empirically underexplored within the context of crime prediction. By evaluating hexagons alongside other unit types across multiple grid sizes and crime types, our study directly responds to Rosser et al.’s (2017) call for broader comparative research on micro-geographic units in predictive crime modelling.

## Data and Methods

### Description of the Study Area and Units of Analysis

**Fig. 1:**
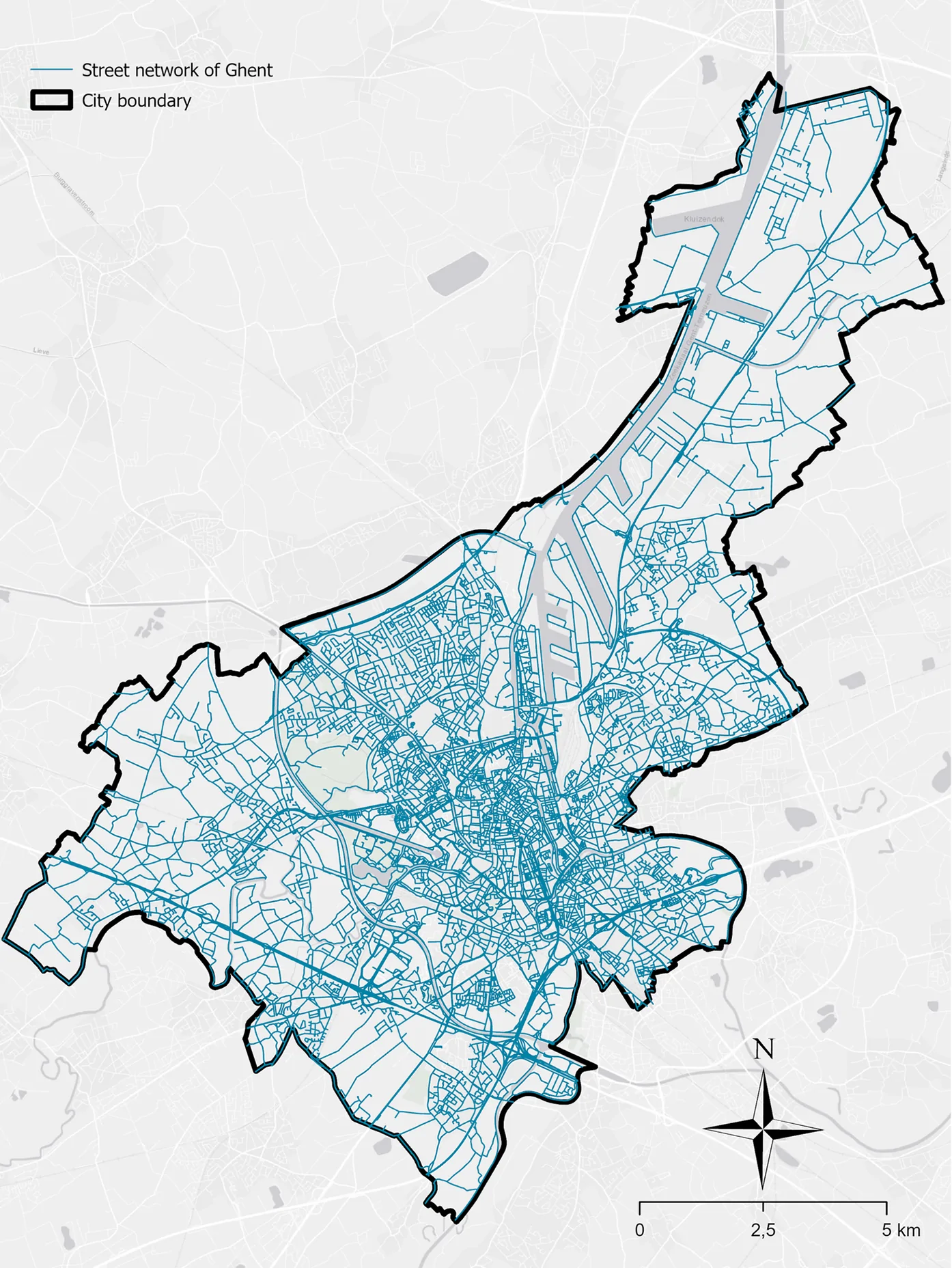
Map of the city of Ghent, its street network and administrative city boundaries

This study is set in Ghent, Belgium’s third-largest city, covering an area of approximately 158 km^2^ and home to 270,473 inhabitants as of 2018. Although Ghent has evolved into a diverse and modern city, its history dates back to the middle ages, which is reflected in its curvilinear street network and historic architecture within the city center (see Fig. 1). Outside the city center, Ghent is characterised by more modern, contemporary neighbourhoods and areas. This mix of both medieval and modern/contemporary spatial lay-out makes Ghent an interesting setting for examining how the use of a specific micro-geographic unit of analysis influences the prediction of spatiotemporal crime patterns.

The shapefile of the street segments was obtained from the City of Ghent’s open data portal, comprising 14,600 unique street segments with an average length of 109 m^1^. In addition to street segments, rectangular and hexagonal grids were created overlaying the study area. Six versions of both the rectangular and hexagonal grid types were created, with cell area resolutions of 0.0025 km², 0.01 km², 0.04 km², 0.09 km², 0.16 km², and 0.25 km². This systematic variation in grid resolution enables us to investigate how changes in the resolution of grid-based units influence the model’s ability to predict crime patterns, also in comparison to the street segment level.

### Data Sources

#### Police-Registered Crime Data

Police-registered crime data were obtained from the Local Police of Ghent spanning the period 2013–2018. The dataset included time, location (raw addresses), and crime type. We selected three crime types, namely residential burglary, aggressive theft, and battery, representing property, mixed, and violent offenses. The raw addresses were geocoded to enable spatial aggregation to the different micro-geographic units.^2^ When exact timestamps were unavailable, we used the midpoint of reported time ranges; events spanning more than seven days were excluded.^3^

Crime records were aggregated spatially to each micro-geographic unit and temporally aggregated into monthly windows, yielding one spatiotemporal observation per spatial unit of analysis per month. These data were used to generate binary target variables (crime/no crime) and crime history features, capturing repeat victimization over fixed and rolling time windows, as well as average crime trends. A monthly prediction resolution was chosen to obtain robust and comparable predictions across the different micro-geographic units of analysis.

#### Data on Crime-Related Risk Factors

In addition to crime data, we included open-source features capturing crime-related risk factors (see Appendix A). To ensure comparability across units, only point- and street-level data were used. Three main data sources were employed.^4^

First, data on various Points of Interest (POIs) from OpenStreetMap were used to model the influence of crime generators, attractors, and detractors (Brantingham & Brantingham, 1995; Cohen & Felson, 1979). Following prior research (Wheeler & Steenbeek, 2021), we calculated both POI density and distance to the nearest POI. For sparsely occurring POIs, we decided to only use the distance to the nearest POI as a feature.

Second, seasonality and weather indicators from OpenWeatherData were added given their assumed impact on crime through routine activity shifts (Corcoran & Zahnow, 2022). For example, certain crimes might increase during specific seasons due to changes in social behaviour or environmental factors (e.g., increased social gatherings during warmer months). Similarly, bad weather conditions might reduce outdoor activities and thereby lower the opportunities for certain types of crime (such as residential burglary), while more favourable weather might have the opposite effect (e.g., battery incidents).

Third, we derived closeness and betweenness centrality measures from Ghent’s street network (transformed into a dual graph), and extrapolated these values to the grid-based units. These centrality measures are commonly applied in criminological research as proxy measures for the level of (human) traffic an area may experience (e.g., Davies & Johnson, 2015; Kim & Hipp, 2020; Mahfoud et al., 2021). Areas with higher closeness or betweenness centrality may experience increased flows of human traffic, which in turn can influence crime opportunity structures. Higher levels of traffic may elevate the likelihood of the spatiotemporal convergence of motivated offenders, suitable targets, and specific levels of (effective) guardianship, core elements of crime opportunity theories (Brantingham & Brantingham, 1995). In addition, these centrality measures may provide a representation of the collective awareness spaces that individuals, including potential offenders, develop through their daily routine activities, rendering these locations potentially more salient in target selection processes as posited by crime pattern theory (Brantingham & Brantingham, 1995; Davies & Johnson, 2015). Hence, while higher centrality may relate to enhanced informal social control through increased visibility (Jacobs, 1961), it may also facilitate crimes that depend on anonymity or target availability. As such, both closeness and betweenness centrality may exert nonlinear effects on crime risk, depending on the specific crime type and local context (Kim & Hipp, 2020; Mahfoud et al., 2021).

### Machine Learning Modelling

The eXtreme Gradient Boosting (XGBoost) algorithm is applied to model the data and derive predictions for each micro-geographic unit and crime type at a monthly prediction resolution.^5^ XGBoost is selected for its flexibility, robustness, and ability to handle complex, non-linear relationships in high-dimensional tabular data (Chen & Guestrin, 2016). Specifically, given the wide range of features proposed in crime and place literature as potentially predictive of crime at specific places and times (e.g., Wheeler & Steenbeek, 2021), XGBoost is well-suited to capture potential non-linear effects and complex interactions among these diverse features (Khalfa et al., 2025).

In general, the XGBoost algorithm is an ensemble tree-based machine learning algorithm, which implies that it fits multiple decision trees collectively to make the predictions rather than relying on a single decision tree. Specifically, XGBoost implements a boosting framework for modelling multiple decision trees, where multiple decision trees are built in a sequential way so that each subsequent decision tree corrects the residual errors made by the ensemble of the previous trees. This iterative process reduces overfitting and controls model complexity (Chen & Guestrin, 2016; James et al., 2013; Zhang et al., 2022).

The prediction datasets, consisting of 56 features for each the micro-geographic units and crime types, were split into 5/6 training data (2013–2017) and 1/6 test data (2018) to predict monthly crime hot spots in 2018. The prediction of the crime hot spots is done using a binary classification task, implying that the XGBoost algorithm is trained to predict the probability of a crime occurring in a specific micro-geographic unit for a specific within a given month in 2018.^6^ This approach is typically referred to as a ‘ranking task’ in machine learning, which implies that all observations are ranked top-down according to their predicted probabilities.

Due to the fact that the training datasets were facing class-imbalance (only few spatiotemporal observations were labelled as crime events), the XGBoost models were trained on resampled training datasets to reduce bias towards the majority class (James et al., 2013). In doing so, a five-fold cross-validation approach was used to tune the most relevant XGBoost hyperparameters and optimise model performance and prevent overfitting.^7^ The maximum depth of each tree was tuned to control the complexity of the trees, ensuring they are not too deep, which could lead to overfitting. Minimum child weight was tuned to prevent the model from learning overly specific patterns by requiring a minimum number of instances in each node before making a split. Subsample ratio and column subsample ratio were also tuned to introduce randomness in the training process, which helps reduce overfitting by ensuring that trees are built using different subsets of data and features. In addition, the learning rate was also set to control the impact of each tree on the final prediction, preventing the model from overfitting by making the updates smaller and more controlled. Finally, the number of estimators was also tuned to balance the number of trees used in the final model, ensuring that enough trees were included to capture patterns without excessively increasing training time or model complexity.

### Model Evaluation

Several performance measures are used to compare the performance of the machine learning models across the different micro-geographic units and crime types. In crime prediction research, performance measures are often calculated based on specific coverage levels (e.g., to account for crime distributions or practical constraints such as limited availability of resources) (Rosser et al., 2017; Rummens & Hardyns, 2020; Wheeler & Steenbeek, 2021). This involves ranking micro-geographic units by their predicted crime probability and determining the proportion of true and false positives and negatives within the top-ranked units up to a defined coverage threshold for a specific temporal window. For grid-based units, this threshold is typically defined as the proportion of the total study area encompassed by the predicted grid cells (area coverage level). For network-based units like street segments, the threshold can correspond to a defined proportion of total network length covered by the predicted segments (network coverage level) (Bowers et al., 2004; Rosser et al., 2017).

However, directly comparing the performance measures across the difference micro-geographic units based on these distinct coverage definitions is problematic. The number of street segments within a single grid cell can vary substantially depending on a grid cell’s location and the street network configuration (Rosser et al., 2017). Comparing model performance across grid-based units using the network coverage level of the grids, by calculating the total street network length intersecting with the selected grid cells, is also problematic, as grid shape and resolution can significantly influence the total length of intersecting streets, undermining meaningful comparisons. To address these challenges, two model evaluation approaches are employed.

First, the prediction performance of models derived for grid-based units (rectangular and hexagonal) are compared to the street segment model by calculating hit rates, precision scores, and F1-scores at a fixed street network coverage level of 5% of the total street network length. This involves selecting the top-ranked grid cells until a cumulative network coverage level of 5% was reached. For the grid-based units, the network coverage level is defined as the total network length intersecting with selected grid cells divided by the total network length. The hit rate quantifies the proportion of correctly predicted hot spots relative to the total number of units where a crime occurred, considering the number of units predicted relative to 5% of the total network length. The precision score quantifies the proportion of correctly predicted hot spots among all units predicted as high-risk by the model according to the 5% network coverage level, indicating the model’s ability to minimise false positives. The F1-score combines hit rate and precision into a single metric (their harmonic mean), offering a balanced evaluation accounting for both false negatives and false positives. The metrics are all calculated across a 12-month average on the test dataset (January-December 2018).

Second, the same performance metrics are used to compare the prediction performance among the grid-based models solely. Hit rates, precision scores, and F1-scores (12-month averages) are computed for both the rectangular and hexagonal grid-based models at a fixed area coverage level of 5% of the total study area. This involves selecting the top-ranked grid cells until a cumulative area coverage of 5% is reached. By fixing the area coverage level, this approach ensures a consistent basis for comparing different grid configurations.

## Results

### Descriptives: Crime Concentration

Prior to presenting the predictive modelling results, we first present descriptive results on the spatial concentration of crime in Ghent. Table 1 summarises the distribution of crime across the rectangular and hexagonal grids, including different grid resolutions, and the street segment level. Two key measures are reported here, namely the percentage of units accounting for 25% and 50% of cumulative crime, and the (generalised) Gini coefficient, which quantifies the proportion of crime within a specific distribution of places. The Gini coefficient ranges from 0 (perfect equality) to 1 (perfect inequality, where all crimes are concentrated in one place) (Bernasco & Steenbeek, 2017). The generalised Gini coefficient is presented in some cases to account for instances where there are more places than crimes, as recommended by Bernasco and Steenbeek (2017).

The results presented in Table 1 show that crime in Ghent is highly concentrated, with aggressive theft and battery incidents exhibiting higher levels of concentration compared to residential burglary across all micro-geographic units of analysis (the highest concentrations are in bold). Looking at the 25% and 50% cumulative proportions of crime, it can be observed that smaller grid-based units of analysis (with a cell area of 0.0025 km²) exhibit the highest crime concentration, with only small differences in crime concentration across different area resolutions of both rectangular and hexagonal grids. Beyond grid resolutions up to 0.01 km², the concentration of crime does not decrease substantially as the resolution decreases, suggesting that further decreases in spatial resolution do not result in substantially lower crime concentrations. At the street segment level, the concentration of crime is also high, but not as high as in small grid-based units of analysis. However, the (generalised) Gini coefficients present a different picture. Especially when compensating for more places than crimes (generalised Gini), crime is most concentrated at 0.04 km² rectangular grids for burglary and battery, while aggressive theft shows highest concentration at 0.09 km² rectangular and hexagonal grids.

**Table 1:** Crime concentration at different micro-geographic units of analysis per type of crime

	Residential burglary			Aggressive theft			Battery incidents		
Unit of analysis	25%	50%	Gini	25%	50%	Gini	25%	50%	Gini
**Rectangular grid cells**									
0.0025 km²	0.59%	2.00%	0.646*	0.07%	0.29%	0.710*	0.08%	0.48%	0.847*
0.01 km²	1.21%	3.79%	0.817*	0.16%	0.73%	0.803*	0.19%	1.11%	0.911*
0.04 km²	1.81%	5.28%	0.837	0.35%	1.39%	0.901*	0.38%	2.09%	0.916
0.09 km²	2.10%	6.00%	0.813	0.41%	1.54%	0.937	0.46%	2.51%	0.897
0.16 km²	2.21%	6.19%	0.803	0.44%	1.68%	0.931	0.53%	2.83%	0.887
0.25 km²	2.41%	6.30%	0.799	0.54%	1.88%	0.926	0.54%	2.95%	0.882
**Hexagonal grid cells**									
0.0025 km²	0.58%	1.97%	0.651*	0.07%	0.28%	0.710*	0.08%	0.48%	0.849*
0.01 km²	1.19%	3.75%	0.820*	0.18%	0.79%	0.798*	0.20%	1.16%	0.910*
0.04 km²	1.73%	5.43%	0.835	0.33%	1.38%	0.902*	0.38%	2.06%	0.914
0.09 km²	2.21%	6.01%	0.814	0.41%	1.59%	0.937	0.46%	2.47%	0.899
0.16 km²	2.42%	6.45%	0.799	0.44%	1.79%	0.929	0.54%	2.78%	0.885
0.25 km²	2.44%	6.37%	0.797	0.41%	1.76%	0.927	0.54%	2.98%	0.880
**Street segments**	1.64%	5.26%	0.780*	0.28%	1.16%	0.739*	0.31%	1.79%	0.874*

*Generalised Gini coefficient

### Prediction Performance of Grid-Based Units vs. Street Segments for Each Type of Crime

Turning to the predictive modelling results, we first present the prediction performance of the machine learning models derived for both the grid-based units of analysis and the street segments at a fixed 5% network coverage level. Table 2 presents the 2018 monthly means and standard deviations of the hit rates, precision scores, and F1-scores for the three types of crime across grid-based units of analysis at varying grid resolutions and street segments at a 5% network coverage level (with the highest values marked in bold).

For residential burglary, it can be observed that the use of street segments to derive monthly burglary predictions generally results in a higher hit rate. Only at higher grid-based resolutions, hit rates are more or less comparable to the hit rate achieved at the street segment level, especially when using hexagonal grid cells ranging from 0.0025 km² to 0.1 km² in grid cell resolution. Importantly, however, is that the use of street segments for predicting residential burglary hot spots results in a large number of false positive predictions at a 5% network coverage level, resulting in overpredicting the number of burglary hot spots. In terms of F1-scores, the best performance is achieved at 5% network coverage level when using rectangular grids with a resolution of 0.16 km² (F1-score = 0.22, on average), which results in a lower number of correctly predicted burglary hot spots compared to street segments, but a higher precision score.

For aggressive theft, higher hit rates are again observed at the street segment level. When predicting for 5% of the total street network length, using street segments as the unit of analysis results in 52.17% of all burglary hot spots being correctly predicted. Hence, the benefits of the street segment approach in terms of hit rate are again evident, but this comes again with the trade-off of a high number of false positives, as the precision score is only 0.98%. When it comes to balancing the performance in terms of both the hit rate and precision of the predictions, the best performance is achieved at the level of hexagonal shaped grids with a resolution of 0.25 km², with an F1-score of 0.39.

Finally, for battery incidents, the highest hit rate is obtained at the level of rectangular grids with a resolution of 0.0025 km², with 43.05% of all battery hot spots correctly predicted at 5% network coverage level on average across 12 prediction months. However, as noted before, a higher hit rate is again associated with lower precision scores, suggesting that predicting at high grid resolutions or at the street segment level, results in a large number of false positive predictions. Based on the average (12-month) F1-scores, the best performance is achieved at the level of rectangular grids with a resolution of 0.04 km² covering 5% of the total network length.

**Table 2:** 2018 means and standard deviations of the monthly prediction performance measures for each crime type across rectangular grids, hexagonal grids and street segments at the 5% network coverage level

	Residential burglary			Aggressive theft			Battery incidents		
Unit of analysis	Hit rate	Precision	F1-score	Hit rate	Precision	F1-score	Hit rate	Precision	F1-score
**Rectangular grid cells**									
0.0025 km²	21.47% (6.58)	1.49% (0.39)	0.03 (0.01)	46.21% (9.31)	0.81% (0.39)	0.02 (0.01)	**43.05% (3.42)**	6.24% (0.92)	0.11 (0.02)
0.01 km²	17.04% (5.61)	4.55% (1.31)	0.07 (0.02)	47.53% (10.63)	3.05% (1.39)	0.06 (0.03)	31.59% (3.04)	15.35% (2.55)	0.21 (0.03)
0.04 km²	15.63% (4.84)	14.51% (3.38)	0.15 (0.04)	45.04% (12.00)	10.61% (5.28)	0.17 (0.08)	24.79% (3.56)	40.79% (4.41)	**0.31 (0.04)**
0.09 km²	13.35% (4.59)	27.40% (7.68)	0.18 (0.06)	41.12% (10.31)	19.23% (8.00)	0.25 (0.08)	18.42% (2.42)	61.60% (7.95)	0.28 (0.05)
0.16 km²	14.53% (4.89)	46.25% (12.33)	**0.22 (0.07)**	34.07% (9.31)	28.84% (11.42)	0.30 (0.10)	14.44% (1.42)	77.85% (9.32)	0.24 (0.02)
0.25 km²	10.96% (3.04)	54.43% (13.61)	0.18 (0.05)	32.36% (13.25)	40.28% (21.27)	0.35 (0.15)	12.05% (1.44)	**88.52% (7.42)**	0.21 (0.02)
**Hexagonal grid cells**									
0.0025 km²	21.06% (5.29)	1.46% (0.27)	0.03 (0.01)	44.99% (14.50)	0.81% (0.39)	0.02 (0.01)	40.55% (2.88)	6.05% (0.79)	0.11 (0.01)
0.01 km²	22.24% (6.58)	5.55% (1.10)	0.09 (0.02)	49.08% (11.65)	3.21% (1.55)	0.06 (0.03)	31.63% (2.30)	15.90% (1.82)	0.21 (0.02)
0.04 km²	17.21% (3.78)	16.33% (2.08)	0.17 (0.03)	44.55% (15.30)	9.96% (4.29)	0.16 (0.06)	23.95% (2.85)	40.74% (6.22)	0.30 (0.04)
0.09 km²	15.04% (4.85)	29.99% (8.23)	0.20 (0.06)	40.35% (11.19)	19.76% (9.64)	0.26 (0.10)	19.41% (1.95)	64.87% (6.76)	0.30 (0.03)
0.16 km²	12.66% (5.25)	39.83% (12.61)	0.19 (0.07)	35.76% (11.06)	30.00% (13.14)	0.32 (0.11)	15.72% (1.78)	82.79% (10.52)	0.26 (0.03)
0.25 km²	12.54% (3.06)	**61.38% (14.41)**	0.21 (0.05)	37.95% (15.09)	**42.31% (18.41)**	**0.39 (0.14)**	12.02% (1.17)	**88.52% (8.00)**	0.21 (0.02)
**Street segments**	**24.03% (5.54)**	3.30% (3.30)	0.06 (0.01)	**52.17% (9.13)**	0.98% (0.48)	0.02 (0.01)	41.51% (3.31)	9.51% (1.11)	0.15 (0.02)

### Prediction Performance Across Rectangular and Hexagonal Grids for Each Type of Crime

Table 3 furthermore summarises the 2018 monthly means and standard deviations of the hit rates, precision scores, and F1-scores for both rectangular and hexagonal grid-based models across the varying grid resolutions and crime types (residential burglary, aggressive theft, and battery). These performance measures are, as previously described, calculated at a 5% area coverage level (with the highest values per unit and performance measure marked in bold).

For residential burglary, increasing the grid resolution is associated with increased hit rates, implying that predicting burglary at smaller grid levels allows for a more accurate identification of burglary hot spots. Nevertheless, across different grid resolutions, the difference in predictive performance between hexagonal and rectangular grid shapes is only minimal, with hexagonal grids only slightly outperforming rectangular grids at both the smallest and largest grid resolutions (0.0025 km² and 0.25 km², respectively). In contrast to the hit rates, the precision of the predictions improves markedly with increasing grid resolutions for both grid types. This suggests that while higher grid resolutions capture a greater number of burglary hot spots, this comes at the expense of generating a higher number of false positive predictions, resulting in over-predicting the number of potential burglary hot spots. In this regard, lower grid resolutions (i.e., larger grid cell areas) mitigate the number of false positive predictions through spatial aggregation. This is also evident when looking at the F1-scores, as a better harmonic balance between both the hit rates and precision scores is achieved at a lower spatial grid resolution, with hexagonal shaped grids slightly outperforming rectangular shaped grids. This shows that that larger grid cells offer a more reliable overall prediction performance despite the reduced spatial sensitivity, irrespective of the grid shape.

For aggressive theft, hit rates are notably higher than those observed for residential burglary but exhibit a similar decreasing trend with decreasing grid resolution. Interestingly, while the hit rates for the hexagonal shaped grid decline consistently with decreasing resolution, rectangular grid hit rates exhibit greater variability across the different resolutions, including a substantial increase at the lowest grid resolution (0.25 km²). In terms of precision, both grid shapes again demonstrate a clear improvement with decreasing grid resolution, reflecting a reduction in false positive predictions while maintaining a sufficient number of true positive predictions, particularly for rectangular grid cells with a grid resolution of 0.25 km². Regarding the F1-scores, the best performance is again achieved at the lowest resolution of 0.25 km², both for rectangular and hexagonal shape grids, yet with the rectangular grids (0.38) slightly outperforming hexagonal grids (0.35) in terms of balancing the hit rate and the precision of the predictions.

Finally, for battery incidents, a higher grid resolution again contributes to an increased percentage of correctly predicted hot spots (hit rate), with rectangular shaped grids only slightly outperforming hexagonal grids across all grid resolutions. The same is true for the precision scores, where rectangular grids again only slightly outperform the hexagonal grids across all grid resolutions. Overall, however, lower spatial resolutions contribute to achieving very high precision scores, thus again introducing less false positive predictions while maintaining a high to moderate hit rate. This is also reflected by the F1-scores, which are generally higher for the models derived for battery incidents compared to the models derived for residential burglary and aggressive theft. In this regard, however, the highest F1-score, and thus balance in terms of hit rate and precision, is now achieved a grid resolution of 0.16 km², with rectangular grids (0.49) again slightly outperforming hexagonal grids (0.48).

**Table 3:** 2018 means and standard deviations of the monthly prediction performance measures for each crime type across rectangular and hexagonal grids at the 5% area coverage level

	Residential burglary			Aggressive theft			Battery incidents		
Unit of analysis	Hit rate	Precision	F1-score	Hit rate	Precision	F1-score	Hit rate	Precision	F1-score
**Rectangular grid cells**									
0.0025 km²	**41.95% (7.71)**	1.08% (0.17)	0.02 (0.03)	**76.01% (9.58)**	0.44% (0.19)	0.01 (0.01)	**66.11% (4.74)**	3.19% (0.45)	0.06 (0.01)
0.01 km²	38.32% (7.23)	3.81% (0.60)	0.07 (0.01)	68.94% (11.77)	1.58% (0.72)	0.03 (0.01)	57.35% (3.25)	10.32% (1.30)	0.18 (0.02)
0.04 km²	35.63% (7.94)	12.91% (2.49)	0.19 (0.04)	66.37% (9.60)	5.79% (2.90)	0.11 (0.05)	48.43% (3.36)	30.40% (3.28)	0.37 (0.03)
0.09 km²	33.01% (9.50)	24.32% (5.55)	0.28 (0.07)	65.91% (3.54)	11.43% (4.72)	0.19 (0.07)	42.11% (3.39)	51.72% (4.81)	0.46 (0.03)
0.16 km²	32.88% (7.36)	38.16% (5.60)	0.35 (0.07)	62.51% (10.68)	17.84% (7.95)	0.27 (0.10)	37.70% (3.38)	69.45% (7.35)	**0.49 (0.04)**
0.25 km²	30.86% (6.23)	**50.68% (7.91)**	**0.38 (0.07)**	68.06% (10.54)	**27.48% (11.22)**	**0.38 (0.12)**	33.21% (3.24)	**81.98% (6.95)**	0.47 (0.04)
**Hexagonal grid cells**									
0.0025 km²	**42.23% (8.01**)	1.08% (0.16)	0.02 (0.01)	**74.70% (8.14)**	0.45% (0.20)	0.01 (0.39)	**65.61% (3.98)**	3.17% (0.47)	0.06 (0.01)
0.01 km²	37.85% (8.23)	3.74% (0.55)	0.07 (0.01)	67.05% (11.99)	1.56% (0.69)	0.03 (0.01)	56.45% (2.35)	10.26% (1.06)	0.17 (0.02)
0.04 km²	34.11% (7.23)	12.40% (1.69)	0.19 (0.03)	67.26% (11.85)	5.77% (2.41)	0.11 (0.04)	47.69% (3.33)	30.10% (3.32)	0.37 (0.03)
0.09 km²	34.41% (7.66)	25.95% (4.71)	0.29 (0.06)	67.93% (9.35)	11.60% (4.27)	0.20 (0.06)	41.79% (3.12)	50.60% (5.17)	0.46 (0.03)
0.16 km²	32.12% (7.68)	38.39% (6.12)	0.35 (0.07)	65.32% (9.26)	19.64% (8.58)	0.30 (0.11)	36.95% (2.18)	68.60% (5.87)	**0.48 (0.03)**
0.25 km²	31.73% (5.89)	**52.03% (6.63)**	**0.39 (0.06)**	62.52% (10.69)	**25.00% (8.56)**	**0.35 (0.09)**	32.59% (3.57)	**79.73% (6.04)**	0.46 0.04)

## Discussion and Conclusion

This study examined the potential of alternative micro-geographic units of analysis compared to the widely used rectangular grid for predicting monthly micro-geographic crime risks using a machine learning (XGBoost) approach. Specifically, this study assessed differences in crime prediction performance across rectangular and hexagonal grids of varying resolutions, as well as street segments, using hit rates, precision scores, and F1-scores as key performance measures. By doing so, this study extends prior research, particularly the work of Rosser et al. (2017), and contributes to the broader field of spatial crime analysis and crime prediction.

Although not the primary focus, our findings confirm that residential burglary, aggressive theft, and battery are spatially concentrated, consistent with the law of crime concentration (Weisburd, 2015; Weisburd et al., 2024). Notably, burglary appeared more spatially dispersed than the other crimes, likely due to its opportunistic nature and more spatially dispersed distribution of target availability.

Street segments proved useful not only for predicting residential burglary, as shown in prior research, but also for predicting aggressive theft and battery incidents. However, while Rosser and colleagues found that street segments outperformed 150 by 150 m rectangular grids in terms of hit rate, our results indicate that when smaller grid cells are used (below an area size of 0.0025 km², approximately 50 × 50 m) the differences in hit rate between street segments and rectangular grids are less pronounced at a 5% network coverage level. Particularly for predicting battery incidents, models based on small rectangular and hexagonal grids performed comparably or better than models based on street segments, suggesting that fine-resolution grids can also capture local crime concentration effectively. While street segments are often viewed as more theoretically meaningful units, our results suggest that this advantage does not automatically translate into better crime prediction performance. However, in contrast to the approach taken by Rosser et al. (2017), we cannot conclude that street segments provide a better representation of how crime risk spreads over the short term, as this was not explicitly modelled throughout our machine learning approach.

Further, the results also show that hexagonal grids can be effectively employed within the context of crime prediction. No substantial differences were observed across rectangular and hexagonal grids, with more or less the same performance observed for both grid types across the different grid resolutions, performance measures and crime types. Their comparable performance suggests that model learning is not strongly influenced by grid geometry. This is an important insight, as it suggests that there is no inherent need to favour rectangular grids over hexagonal grids when employing grid-based crime predictions, particularly if there are specific assumptions beyond prediction performance for adopting hexagonal grids instead of rectangular grids within the context of predicting micro-geographic crime risks (e.g., reduced edge effects, uniform connectivity, and improved spatial visualization).

In addition, the results of this study further confirm that when grid-based units of analysis are used, crime prediction performance generally declines as the spatial resolution increases. While smaller rectangular and hexagonal grids yielded higher hit rates, a more balanced prediction performance was observed at lower grid resolutions. This aligns with previous research (e.g., Lin et al., 2018; Rummens & Hardyns, 2021), but extends these existing insights by demonstrating that the effect of grid resolution on prediction performance is consistent for both rectangular and hexagonal grid types.

One plausible explanation for the observed differences in prediction performance across micro-geographic units may lie in the varying sample sizes of the potential high-risk locations/units of analysis. Street segments or fine-grained grids yield a larger pool of candidate hot spots compared to coarser grids, thereby increasing the likelihood of correctly identifying high-risk areas due to the concentrated nature of crime. For instance, at a 5% network coverage level, models applied to numerous small units (e.g., 14,600 street segments) will likely classify more high-risk units than models applied to fewer large grids (e.g., 700 cells), providing more opportunities to capture crime occurrences. However, this increased sensitivity also introduces more false positives, as a greater number of units may be misclassified as high-risk.

Lastly, this study also showed some notable differences in prediction performance across crime types. Monthly predictions for battery incidents yielded relatively higher and more stable performance metrics, likely due to high spatial concentration of battery incidents around social interaction nodes and consistent environmental patterns. In contrast, residential burglary, despite comparable frequencies to battery incidents, showed lower prediction performance, possibly reflecting a more dispersed spatial distribution and greater variability in offender decision-making. These findings align with prior research indicating that battery incidents are generally more spatially stable and predictable compared to residential burglaries (e.g., Khalfa et al., 2023; Rummens et al., 2017). For aggressive theft, despite high spatial concentration, lower model precision and increased false positives were observed, likely due to its lower prevalence and subsequent overprediction of crime hot spots.

### Limitations of the Study

Some important limitations should be considered when interpreting the results and findings of this study:

First, the geographic scope of this study is restricted to a single urban setting, namely Ghent (Belgium). Consequently, the generalizability of the findings to other, particularly US-based, settings is limited. Notwithstanding this limited generalizability, the contribution of this study to the European context may be substantial, given the relative dearth of research on crime prediction within European settings and contexts. However, it is clear that further research is also needed to corroborate our findings within alternative settings and contexts.

Second, this study employed one specific machine learning algorithm (XGBoost) to derive monthly predictions of crime across micro-geographic units of analysis. Although the XGBoost algorithm provides a robust machine learning algorithm for modelling spatiotemporal crime patterns, exploring other algorithms or modelling approaches could have yielded different results.

Third, this study focused on deriving monthly crime predictions and did not further explore the sensitivity of the results across different temporal prediction resolutions. However, as demonstrated by prior research, increasing the temporal resolution to, for example, bi-weekly, weekly, or daily prediction resolutions would further partition the data even more, potentially leading to reduced model performance due to data sparsity, class imbalance and increased noise.

Fourth, while a comprehensive set of features was employed as input to the machine learning models, the number of features includes was also constrained by data availability at the point or street level. For example, due to the absence of socio-demographic and socio-economic data at such a fine-grained spatial scale, this study did not incorporate features related to social disorganization. Furthermore, the study did not explore the use of other, more fine-grained spatiotemporal big data sources on relevant crime predictors (e.g., the ambient population) due to the limited availability and accessibility of such data sources over extended time periods at fine-grained spatial scales.

Finally, in evaluating model performance across micro-geographic units of analysis, this study focused on predictions at a fixed coverage level of 5%, both in terms of area and network coverage. While this fixed threshold approach provides a standardised basis for comparison, it does not explore the full spectrum of trade-offs between precision and recall across varying coverage thresholds. Although prior research suggests that varying the prediction threshold does not typically result in substantial differences in relative model performance across different units of analysis, examining performance across different threshold could have provided an even more comprehensive evaluation of model performance.

### Implications and Recommendations for Future Research and Policy

This study shows that, beyond the commonly used rectangular grid, street segments and hexagonal grids may provide viable micro-geographic units of analysis for predicting micro-geographic crime risks using machine learning. However, several implications arise for future research and policy:

**Model evaluation and unit selection.** More attention should be paid to how model performance is defined and operationalised when predicting crime risks at micro-geographic levels, as well as to the implications of using specific units of analysis for crime prediction. This is particularly important from a policy perspective, given that crime prediction models are increasingly used to guide the allocation of proactive policing and crime prevention efforts. The choice of spatial unit and performance metrics influences not only the perceived effectiveness of models but also their practical utility in operational settings. For instance, deploying police resources to street segments may be more feasible and efficient than targeting small grid cells, as segments often align better with natural patrol routes and allow for more concrete, actionable instructions (Rosser et al., 2017). However, although street segments often produce higher hit rates, they also tend to generate more false positives, potentially leading to the deployment of resources in areas that are not actually at risk, potentially introducing net widening effects.

**Spatial granularity and prediction error.** Further, it is important to recognise that at very small spatial scales, such as very small grid cells or street segments, the operational significance of prediction error may be less clear-cut. What is formally a false positive may simply be a (minor) spatial displacement that does not reduce the practical value of the prediction for resource deployment. Such deviations may thus also reflect spatial noise rather than true model failure. In these cases, near-location predictions can still be useful, especially when considering the diffusion of crime prevention benefits (e.g., Braga et al., 2019). This may highlight the need to consider spatially tolerant performance metrics (such as the near hit rate) that account for spatial proximity between predicted and observed hot spots in reflecting true/false positive/negative predictions. However, targeting very small areas can also lead to operational inefficiencies, such as increased patrol boredom, underscoring the importance of aligning spatial granularity with intervention feasibility. In this regard, future research should also examine how practitioners interpret and apply risk maps based on specific spatial units of analysis. Whether hot spots are visualised via grid-based approaches or street segments may potentially affect understanding, trust and specific perceptions regarding crime risk predictions. Insights into how practitioners engage with different visual representations can inform the design of more intuitive, actionable tools for crime prevention.

**Hybrid approaches.** An important question is also whether a more spatially hybrid prediction approach could enhance prediction performance in terms of both hit rate and precision (e.g., F1-score). Current prediction models typically employ a single micro-geographic unit of analysis (e.g., grid cells of fixed size), but combining predictions across different units may potentially yield more optimised results. A promising direction for future research and policy is to develop hybrid models that integrate predictions from multiple unit types while maintaining a consistent area or network coverage level. This could involve merging non-overlapping high-risk areas into a single output to capture a broader range of hot spots. In case of overlap, a weighting scheme based on individual model performance could be used to prioritise or combine the predictions.

**Model complexity vs. model interpretability**. Another important focus should be on examining the trade-off between model complexity and model interpretability. While future research should definitely further explore the potential of non-parametric machine learning models and the integration of fine-grained spatiotemporal data such as mobile phone, social media, or police GPS data to improve and optimise crime prediction models, it will be equally important to consider how this increased model complexity affects model interpretability and the extent to which practitioners can understand, trust, and apply crime risk predictions. In this regard, future work should invest in the (further) development and application of Explainable AI (XAI) methods to enhance the interpretability of complex crime prediction models. By providing clear insights into the contribution of specific predictors and the rationale behind risk classifications made by machine learning models, XAI can help bridge the gap between technical model outputs and practical decision-making. This is especially important in crime prevention, where interpretability is important for designing effective and accountable interventions based on model predictions (see e.g., Khalfa et al., 2025 for an application of XAI in relation to crime prediction).

**Evaluation of real-world effects.** Finally, more evaluation research is needed to assess the impact of micro-geographic crime predictions in practice. Does the use of spatiotemporal crime predictions lead to measurable crime reduction or prevention effects when used to allocate targeted patrols or crime prevention efforts? The spatial unit matters here too: using very small spatial scales (such as very small grids) may reduce statistical power due to low crime counts, as shown by Taylor and Ratcliffe (2020). Larger units may potentially offer better conditions for detecting intervention effects in experimental settings.

### Conclusion

While rectangular grids remain the dominant micro-geographic unit of analysis in crime prediction, this study shows that street segments and hexagonal grids may offer viable alternatives, each with distinct strengths and limitations in terms of prediction performance and practical utility. Advancing crime prediction research and practices will require a more hybrid and critically informed approach to spatial unit selection, one that carefully balances the trade-offs between prediction performance, operational feasibility, and ethical implications.

## Electronic Supplementary Material

Below is the link to the electronic supplementary material.

Supplementary Material 1

## Data Availability

No datasets were generated or analysed during the current study.

## Footnotes

1 See: https://data.stad.gent/explore/dataset/wegsegmenten-gent/table/.

2 The geocoding process was primarily done using the official address reference database of the Flemish government. In cases where addresses could not be geocoded using this primary method, the ArcGIS geocoder API in Python was used. Incidents missing exact house numbers were geocoded to the middle of the corresponding street.

3 This was only the case for less than 5% of the total number of crimes for each crime type. The total number of crimes used for analysis are the following; 10,186 (residential burglary); 2,234 (aggressive theft); and 9,114 (battery incidence).

4 As the environmental and temporal features in our study were engineered separately for 13 different spatial unit configurations, varying by both unit type and resolution, providing full descriptive statistics for each configuration would exceed the scope and length constraints of the manuscript. Therefore, summary statistics (e.g., mean, variance, spatial coverage) of the key features are available upon request from the corresponding author.

5 All analyses were conducted using Python, version 3.12.

6 Hence, the target variable in this study involves a dichotomized variable: no crime vs. crime.

7 For an overview of the most relevant XGBoost hyperparameters, see: https://xgboosting.com/most-important-xgboost-hyperparameters-to-tune/.

## References

[CR5] Bernasco, W., & Steenbeek, W. (2017). More places than crimes: Implications for evaluating the law of crime concentration at place. *Journal of Quantitative Criminology*, *33*, 451–467.

[CR6] Birch, C. P., Oom, S. P., & Beecham, J. A. (2007). Rectangular and hexagonal grids used for observation, experiment and simulation in ecology. *Ecological Modelling*, *206*(3–4), 347–359.

[CR7] Bowers, K., & Johnson, S. D. (2014). Crime mapping as a tool for security and crime prevention. *The Handbook of Security*, 566–587.

[CR8] Bowers, K. J., Johnson, S. D., & Pease, K. (2004). Prospective hot-spotting: The future of crime mapping? *British Journal of Criminology*, *44*(5), 641–658.

[CR1] Braga, A. A., Turchan, B. S., Papachristos, A. V., & Hureau, D. M. (2019). Hot spots policing and crime reduction: An update of an ongoing systematic review and meta-analysis. *Journal of Experimental Criminology*, *15*, 289–311.

[CR2] Brantingham, P., & Brantingham, P. (1995). Criminality of place: Crime generators and crime attractors. *European Journal on Criminal Policy and Research*, *3*, 5–26.

[CR3] Brantingham, P., & Brantingham, P. (2013). Crime pattern theory. In *Environmental criminology and crime analysis* (pp. 100–116). Willan.

[CR4] Brantingham, P. L., Brantingham, P. J., Vajihollahi, M., & Wuschke, K. (2009). Crime analysis at multiple scales of aggregation: A topological approach. *Putting Crime in its Place: Units of Analysis in Geographic Criminology*, 87–107.

[CR9] Chen, T., & Guestrin, C. (2016, August). Xgboost: A scalable tree boosting system. In *Proceedings of the 22nd acm sigkdd international conference on knowledge discovery and data mining* (pp. 785–794).

[CR10] Cohen, L. E., & Felson, M. (1979). Social change and crime rate trends: A routine activity approach. *American Sociological Review*, *44*(4), 588–608.

[CR11] Corcoran, J., & Zahnow, R. (2022). Weather and crime: A systematic review of the empirical literature. *Crime Science*, *11*(1), 16.

[CR12] Davies, T., & Johnson, S. D. (2015). Examining the relationship between road structure and burglary risk via quantitative network analysis. *Journal of Quantitative Criminology*, *31*, 481–507.

[CR13] Eck, J., & Weisburd, D. (1995). Crime places in crime theory. *Crime and Place: Crime Prevention Studies*, *4*, 1–33.

[CR14] Evans, D., & Herbert, D. (1989). *The geography of crime*. Routledge.

[CR15] Hardyns, W., & Rummens, A. (2018). Predictive policing as a new tool for law enforcement? Recent developments and challenges. *European Journal on Criminal Policy and Research*, *24*, 201–218.

[CR16] Harinam, V., Bavcevic, Z., & Ariel, B. (2022). Spatial distribution and developmental trajectories of crime versus crime severity: Do not abandon the count-based model just yet. *Crime Science*, *11*(1), 14.

[CR17] Hart, T. C. (2020). Hot spots of crime: Methods and predictive analytics. *Geographies of Behavioural Health Crime and Disorder: the Intersection of Social Problems and Place*, 87–103.

[CR18] Hoet, M. J. (2023). Crime concentration and hot spot dynamics: An examination of homicides in Santa fe, Argentina. *International Criminology*, *3*(4), 313–327.

[CR46] Jacobs, J. (1961). *The life and death of Great American cities*. Random House.

[CR19] James, G. (2013). An introduction to statistical learning.

[CR20] Johnson, S. D. (2010). A brief history of the analysis of crime concentration. *European Journal of Applied Mathematics*, *21*(4–5), 349–370.

[CR21] Johnson, S. D., & Bowers, K. J. (2004). The burglary as clue to the future: The beginnings of prospective hot-spotting. *European Journal of Criminology*, *1*(2), 237–255.

[CR22] Johnson, S. D., & Bowers, K. J. (2010). Permeability and burglary risk: Are cul-de-sacs safer? *Journal of Quantitative Criminology*, *26*, 89–111.

[CR23] Kadar, C., Maculan, R., & Feuerriegel, S. (2019). Public decision support for low population density areas: An imbalance-aware hyper-ensemble for spatio-temporal crime prediction. *Decision Support Systems*, *119*, 107–117.

[CR24] Khalfa, R., Snaphaan, T., Pauwels, L., Kounadi, O., & Hardyns, W. (2023). Crime within a bandwidth: Testing the law of crime concentration at place in Brussels. *European Journal on Criminal Policy and Research*, 1–28.

[CR25] Khalfa, R., Theinert, N., & Hardyns, W. (2025). *Comparing XAI techniques for interpreting short-term burglary predictions at micro-places*. Computational Urban Science. 10.1007/s43762-025-00185-x PMC12064634 40352344

[CR26] Kim, Y. A., & Hipp, J. R. (2020). Pathways: Examining street network configurations, structural characteristics and Spatial crime patterns in street segments. *Journal of Quantitative Criminology*, *36*, 725–752.

[CR27] Kounadi, O., Ristea, A., Araujo, A., & Leitner, M. (2020). A systematic review on Spatial crime forecasting. *Crime Science*, *9*, 1–22. 10.1186/s40163-020-00116-7 PMC7319308 32626645

[CR28] Kronkvist, K., Borg, A., Boldt, M., & Gerell, M. (2025). Predicting public violent crime using register and openstreetmap data: A risk terrain modeling approach across three cities of varying size. *Applied Spatial Analysis and Policy*, *18*(1), 1–34.

[CR29] Lin, Y. L., Yen, M. F., & Yu, L. C. (2018). Grid-based crime prediction using geographical features. *ISPRS International Journal of Geo-Information*, *7*(8), 298.

[CR30] Mahfoud, M., Bernasco, W., Bhulai, S., & van der Mei, R. (2021). Forecasting spatio-temporal variation in residential burglary with the integrated Laplace approximation framework: Effects of crime generators, street networks, and prior crimes. *Journal of Quantitative Criminology*, *37*, 835–862.

[CR31] Ohyama, T., & Amemiya, M. (2018). Applying crime prediction techniques to japan: A comparison between risk terrain modeling and other methods. *European Journal on Criminal Policy and Research*, *24*, 469–487.

[CR32] Prieto Curiel, R. (2023). Weekly crime concentration. *Journal of Quantitative Criminology*, *39*(1), 97–124. 34483469 10.1007/s10940-021-09533-6 PMC8408821

[CR33] Rosser, G., Davies, T., Bowers, K. J., Johnson, S. D., & Cheng, T. (2017). Predictive crime mapping: Arbitrary grids or street networks? *Journal of Quantitative Criminology*, *33*, 569–594. 32025086 10.1007/s10940-016-9321-x PMC6979510

[CR34] Rummens, A., & Hardyns, W. (2020). Comparison of near-repeat, machine learning and risk terrain modeling for making Spatiotemporal predictions of crime. *Applied Spatial Analysis and Policy*, *13*(4), 1035–1053.

[CR35] Rummens, A., & Hardyns, W. (2021). The effect of Spatiotemporal resolution on predictive policing model performance. *International Journal of Forecasting*, *37*(1), 125–133.

[CR36] Rummens, A., Hardyns, W., & Pauwels, L. (2017). The use of predictive analysis in Spatiotemporal crime forecasting: Building and testing a model in an urban context. *Applied Geography*, *86*, 255–261.

[CR38] Sherman, L. W., & Weisburd, D. (1995). General deterrent effects of Police patrol in crime hot spots: A randomized, controlled trial. *Justice Quarterly*, *12*(4), 625–648.

[CR37] Sherman, L. W., Gartin, P. R., & Buerger, M. E. (1989). Hot spots of predatory crime: Routine activities and the criminology of place. *Criminology*, *27*(1), 27–56.

[CR39] Taylor, R. B., & Ratcliffe, J. H. (2020). Was the Pope to blame? Statistical powerlessness and the predictive policing of micro-scale randomized control trials. *Criminology & Public Policy*, *19*(3), 965–996.

[CR40] Weisburd, D. (2015). The law of crime concentration and the criminology of place. *Criminology*, *53*(2), 133–157.

[CR41] Weisburd, D., Bernasco, W., & Bruinsma, G. (Eds.). (2009). *Putting crime in its place*. Springer.

[CR42] Weisburd, D., Groff, E. R., & Yang, S. M. (2012). *The criminology of place: Street segments and our Understanding of the crime problem*. Oxford University Press.

[CR44] Weisburd, D., Zastrow, T., Kuen, K., & Andresen, M. (2024). Crime concentrations at micro places: A review of the evidence. *Aggression and Violent Behavior*, 101979.

[CR43] Wheeler, A. P., & Steenbeek, W. (2021). Mapping the risk terrain for crime using machine learning. *Journal of Quantitative Criminology*, *37*, 445–480.

[CR45] Zhang, X., Liu, L., Lan, M., Song, G., Xiao, L., & Chen, J. (2022). Interpretable machine learning models for crime prediction. *Computers Environment and Urban Systems*, *94*, 101789.

