# Supplementary material for: Comparing Machine Learning-Based Crime Predictions Across Micro-Geographic Units: Street Segments, Rectangular Grids, and Hexagonal Grids
Source: Appl Spat Anal Policy. 2025 Jun 25;18(3):79. doi: 10.1007/s12061-025-09683-1 (PMC13578039; doi:10.1007/s12061-025-09683-1)
Supplement: Supplementary file 1 [file 12061_2025_9683_MOESM1_ESM.docx]

**Appendix A: Features included in the machine learning modelling**

| **Type** | **Name** | **Description** | **Source** |
| --- | --- | --- | --- |
| Crime | lag_1m,  lag_2m,  lag_3m,  lag_4m,  lag_6m,  lag_1y, | Number of offenses recorded in a grid cell during specific fixed time lags relative to the prediction date (e.g., lag_2m indicates the number of offenses that occurred exactly two months before the prediction date) | Local police of Ghent (Belgium) |
|  | roll_1m,  roll_2m,  roll_3m,  roll_4m,  roll_6m,  roll_1y, | Cumulative number of offenses recorded in a grid cell over a continuous time window ending at the prediction date (e.g., roll_2m sums up the total offenses from the last two months leading up to the prediction date) |  |
|  | average_3m,  average_6m,  average_9m,  average_1y | Average number of offenses recorded in a grid cell over a specified time period (e.g., average_6m calculates the mean number of offenses in the six months preceding the prediction date) |  |
| Environmental characteristics | green | Presence of green area (Boolean) | OpenStreetMap |
|  | banks | Number of banks |  |
|  | tourist_acc | Number of tourist accommodations |  |
|  | bars_nightclubs | Number of bars and nightclubs |  |
|  | restaurants | Number of restaurants |  |
|  | cafes | Number of cafes |  |
|  | convenience | Number of convenience stores |  |
|  | education | Number of educational facilities |  |
|  | fast_food | Number of fast food restaurants |  |
|  | grocery | Number of grocery stores |  |
|  | sport_facil | Number of sport facilities |  |
|  | shops | Number of shops (other than convenience and grocery stores) |  |
|  | residential_units | Number of residential units |  |
|  | dist_banks | Distance to the nearest bank |  |
|  | dist_tourist_acc | Distance to the nearest tourist accommodation |  |
|  | dist_bars_nightclubs | Distance to the nearest bar or nightclub |  |
|  | dist_rest | Distance to the nearest restaurant |  |
|  | dist_cafe | Distance to the nearest cafe |  |
|  | dist_convenience | Distance to the nearest convenience store |  |
|  | dist_education | Distance to the nearest educational facility |  |
|  | dist_fast_food | Distance to the nearest fast food restaurant |  |
|  | dist_grocery | Distance to the nearest grocery store |  |
|  | dist_sport_facil | Distance to the nearest sport facility |  |
|  | dist_shops | Distance to the nearest shop (other than convenience and grocery store) |  |
|  | dist_fire | Distance to the nearest fire station |  |
|  | dist_hospital | Distance to the nearest hospital |  |
|  | dist_police_station | Distance to the nearest police station |  |
|  | dist_public_building | Distance to the nearest public building (excluding fire and police stations and hospitals) |  |
|  | dist_train_station | Distance to the nearest train station |  |
|  | dist_bus_stop | Distance to the nearest bus stop |  |
|  | dist_tram_stop | Distance to the nearest tram stop |  |
|  | dist_highway | Distance to the nearest highway |  |
|  | betweenness_cent | Betweenness centrality |  |
|  | closeness_cent | Closeness centrality |  |
| Temporal | temp | Mean temperature | OpenWeatherData |
|  | dew_point | Mean dew point |  |
|  | pressure | Mean air pressure |  |
|  | wind_speed | Mean wind speed |  |
|  | humidity | Mean humidity |  |
